# Supplementary material for: The interactome of intact mitochondria by cross-linking mass spectrometry provides evidence for coexisting respiratory supercomplexes
Source: Mol Cell Proteomics. 2017 Dec 8;17(2):216–32. doi: 10.1074/mcp.RA117.000470 (PMC5795388; doi:10.1074/mcp.RA117.000470)
Supplement: Supplemental Data [file supp_RA117.000470_133922_0_supp_23978_fzffwf.zip › spectra_annotation/mito_spectra_annotation/308-1-10-1-10-1.pdf]

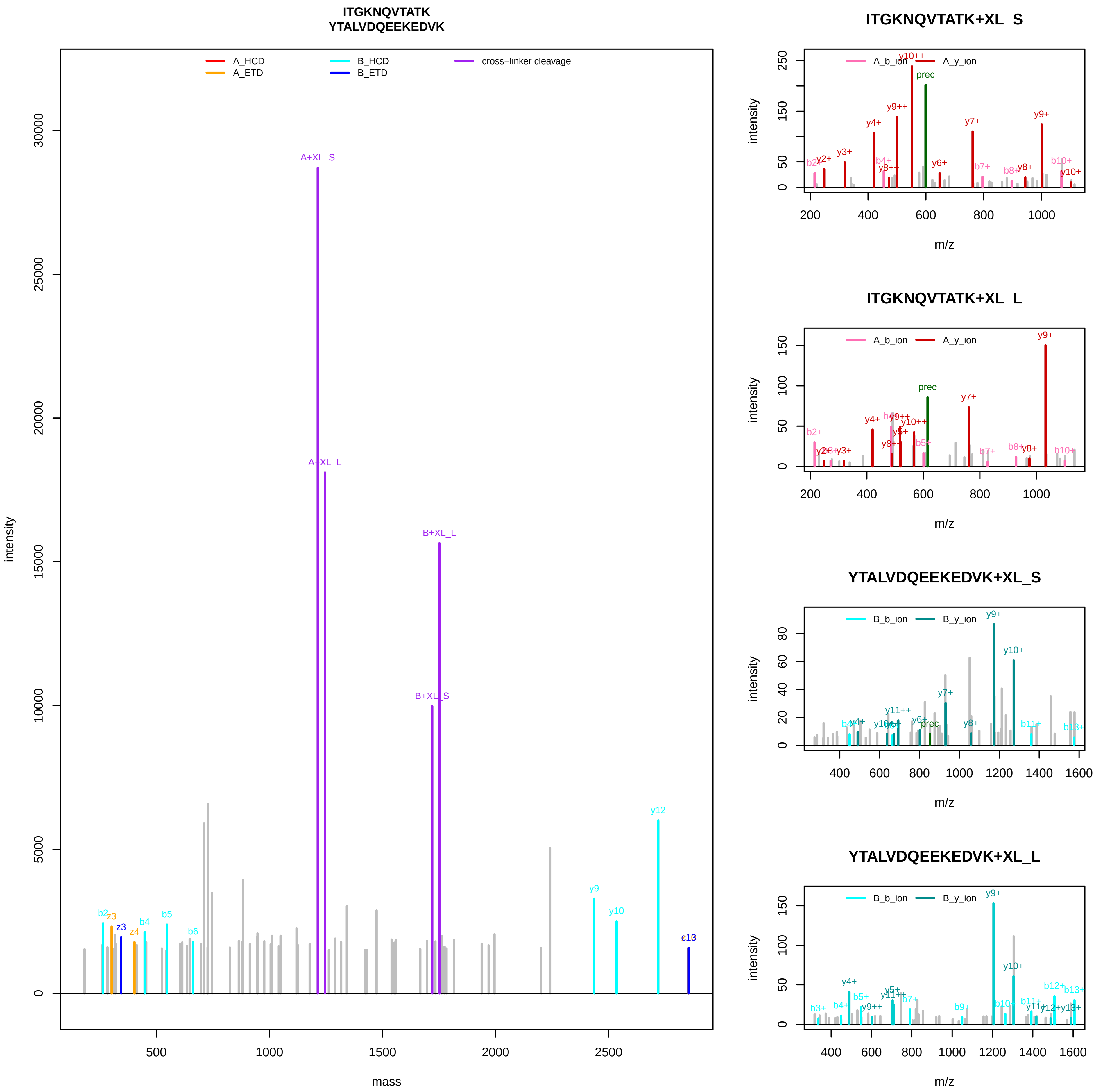

ITGKNQVTATK
YTALVDQEEKEDVK
A_HCD
A_ETD
B_HCD
B_ETD
cross−linker cleavage
A+XL_S
A+XL_L
B+XL_L
B+XL_S
b2
z3
z3
z4
b4
b5
b6
y9
y10
y12
c13
intensity
mass
0
5000
10000
15000
20000
25000
30000
500
1000
1500
2000
2500
ITGKNQVTATK+XL_S
A_b_ion
A_y_ion
y10++
prec
y9++
y4+
y3+
y2+
b2+
b4+
y8++
y6+
y7+
b7+
b8+
y8+
y9+
b10+
y10+
ITGKNQVTATK+XL_L
YTALVDQEEKEDVK+XL_S
B_b_ion
B_y_ion
y11++
y7+
y10+
y8+
b11+
b13+
YTALVDQEEKEDVK+XL_L
y4+
b3+
b4+
b5+
y9++
y5+
y11++
b7+
b9+
b10+
b11+
y11+
y12+
y13+
b12+
b13+
m/z
